# Significance of serum ferritin as a prognostic factor in advanced hepatobiliary cancer patients treated with Korean medicine: a retrospective cohort study

**DOI:** 10.1186/s12906-018-2240-7

**Published:** 2018-06-07

**Authors:** Anna Song, Wankyu Eo, Sehyun Kim, Bumsang Shim, Sookyung Lee

**Affiliations:** 1 Department of Clinical Korean Medicine, Graduate School, Kyung Hee University, Seoul, South Korea; 2 Department of Medical Oncology and Hematology, College of Medicine, Kyung Hee University, Seoul, South Korea; 3 Graduate School, Dankook University, Yongin, South Korea; 4 Department of Pathology, College of Korean Medicine, Kyung Hee University, Seoul, South Korea; 5 Department of Clinical Oncology, College of Korean Medicine, Kyung Hee University Hospital at Gangdong, 892 Dongnam-ro, Gangdong-gu, Seoul, 05278 Republic of Korea

**Keywords:** Hepatocellular carcinoma, Biliary tract neoplasms, Survival, Prognosis, Ferritins

## Abstract

**Background:**

Advanced hepatobiliary cancers are highly lethal cancers that require precise prediction in clinical practice. Serum ferritin level increases in malignancy and high serum ferritin level is associated with poor survival in various cancers. This study aimed to identify whether serum ferritin could independently predict the overall survival (OS) of patients with advanced hepatobiliary cancers.

**Methods:**

The retrospective cohort study was performed by reviewing medical records of patients with advanced hepatobiliary cancers from June 2006 to September 2016. The demographic and clinicopathological characteristics as well as the biochemical markers were evaluated at the initiation of Korean medicine (KM) treatment. The OS was calculated using Kaplan-Meier estimates. The Cox proportional hazard model was used to identify the independent prognostic significance of serum ferritin for survival.

**Results:**

The median OS of all subjects was 5.1 months (range, 0.5–114.9 months). The median OS of group with low ferritin levels and that with high ferritin levels was 7.5 months (range, 0.7–114.9 months) and 2.8 months (range, 0.5–22.8 months), respectively (*P* < 0.001). The results of the univariate analysis showed that the Eastern Cooperative Oncology Group Performance Status (ECOG-PS) (*P* = 0.002), tumor type (*P* = 0.001), prior treatment (*P* = 0.023), serum ferritin (*P* < 0.001), hemoglobin (*P* = 0.002), total bilirubin (*P* = 0.002), gamma-glutamyl transpeptidase (*P* = 0.007), albumin (*P* = 0.013), white blood cell (*P* = 0.002), and C-reactive protein (CRP) (*P* < 0.001) were significant factors for the patients’ survival outcome. On multivariate analysis controlling confounding factors, ferritin (*P* = 0.041), CRP (*P* = 0.010), ECOG-PS (*P* = 0.010), and tumor type (*P* = 0.018) were identified as independent prognostic factors for survival.

**Conclusions:**

These results indicate that serum ferritin is a valid clinical biochemical marker to predict survival of patients with advanced hepatobiliary cancers.

## Background

Hepatobiliary cancers, including hepatocellular carcinoma (HCC) and biliary tract cancers (BTC) are often incurable and highly lethal cancers. The BTC are classified as either cholangiocarcinoma of the bile duct or gall bladder cancer, according to the site of the tumor. In addition, HCC is the fourth most common cancer and the second leading cause of cancer death in South Korea [1]. Although the incidence of BTC is relatively low, the heavy disease burden is associated with poor survival outcome. Even if BTC is diagnosed at an early stage and curative resection is performed, a short recurrence-free survival (11–20 months) and a high recurrence rate (53–62%) have been reported in previous studies [2–5]. Furthermore, most patients are initially diagnosed with advanced stage hepatobiliary cancer [6].

Patients with advanced hepatobiliary cancers have limited systemic chemotherapy options: sorafenib for advanced HCC and cisplatin plus gemcitabine for advanced BTC [7–9]. Unlike other types of cancer, the progression of hepatobiliary cancers is deeply related to liver function. Progression of tumor causes deterioration of the liver function, but repeated treatment can also damage liver function; thus, selecting an appropriate treatment modality is difficult in clinical practice. For a decade, patients with advanced hepatobiliary cancers have had limited standard treatment options and most of them underwent anticancer therapy with limited or only short-term survival gain.

To provide optimized treatment for patients, precise survival prediction is required in clinical practice. Identifying indicators that can predict the survival outcome of patients with advanced hepatobiliary cancers will help practitioners provide optimal care and management of these patients. According to recent studies, Child-Pugh score, body mass index (BMI), and aspartate aminotransferase (AST) for HCC and Eastern Cooperative Oncology Group Performance Status (ECOG-PS), carcinoembryonic antigen, carbohydrate antigen 19–9, hemoglobin (Hb), white blood cell (WBC), and albumin for BTCs have been identified as potential prognostic biomarkers for predicting survival in advanced hepatobiliary cancers [2, 10, 11].

Ferritin is an iron storage protein that is abundant in intracellular compartments. A small amount of ferritin also exists in blood and is called serum ferritin. Serum ferritin is a surrogate marker of stored iron, and 1 ng/mL of serum ferritin is equal to 8 mg of stored iron [12]. The level of serum ferritin tends to increase with age and is relatively higher in men than in women [13]. Elevated serum ferritin often indicates iron over-load, but it also increases in inflammation, liver disease, and malignancy [14–19]. Previous studies have reported that high serum ferritin is associated with poor prognosis in pancreatic cancer, colorectal cancer, lung cancer, peripheral T-cell lymphoma, and HCC [16–20]. However, the prognostic significance of serum ferritin for survival in patients with advanced hepatobiliary cancers has not been investigated.

Therefore, this study was performed to identify the potential prognostic impact of serum ferritin level in predicting survival in patients with advanced hepatobiliary cancers treated with Korean medicine (KM).

## Methods

This study was based on a retrospective cohort design and approved by the Institutional Review Board of Kyung Hee University Hospital at Gangdong (KHNMC-OH-IRB 2016–10-006).

### Study subjects

For this study, we reviewed medical records of patients with hepatobiliary cancers from June 2006 to September 2016. Inclusion criteria were as follows: patients with hepatobiliary cancer classified as stage IV disease according to the American Joint Committee on Cancer Tumor Node Metastasis staging (7th ed., 2010), those aged > 20 years treated with KM monotherapy, those who had a serum ferritin value measured before the treatment, and those who had an ECOG-PS of 0–2.

**Fig. 1:**
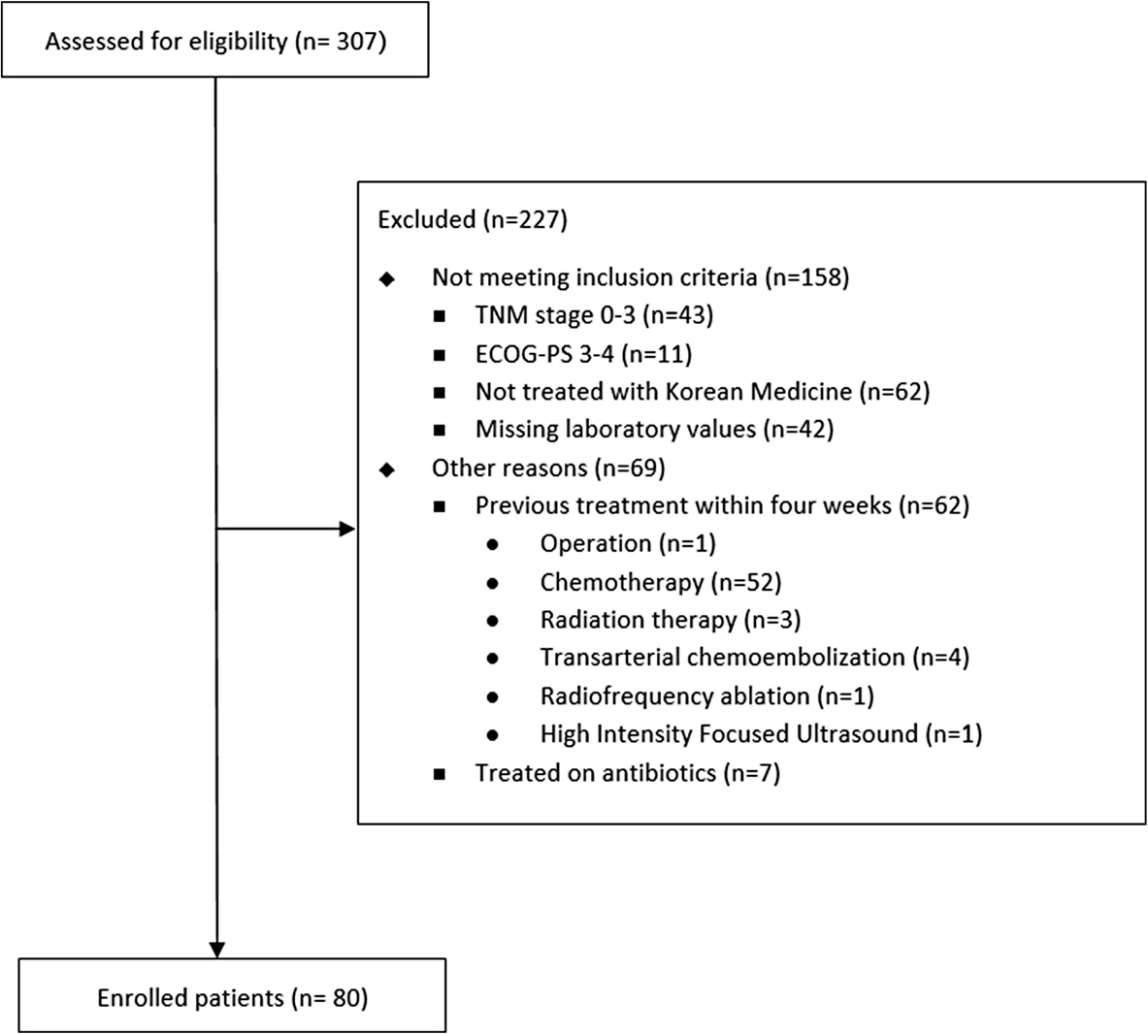
Flow chart of status of patients with advanced hepatobiliary cancers

Patients who were concurrently treated with KM combined with any conventional cancer therapy, such as transarterial chemoembolization, radiofrequency ablation (RFA), high-intensity focused ultrasound, surgery, chemotherapy, and radiotherapy, were excluded in this study to eliminate the effects of conventional cancer therapy. In addition, patients who had undergone conventional cancer treatment within 4 weeks from the initial laboratory tests were excluded to avoid the effects of conventional treatment on the biochemical markers. At the time of laboratory testing, patients with signs of inflammation or those treated with antibiotics were also excluded to rule out the influence induced by inflammation. (Fig. 1).

### Demographic, clinicopathological, and biochemical factors

Patient demographic and clinicopathological characteristics and biochemical markers were investigated. Demographic and clinicopathologic characteristics included age, gender, ECOG-PS, BMI, tumor type, and prior treatments. Biochemical markers, including serum ferritin, Hb, total bilirubin, AST, alanine aminotransferase (ALT), gamma-glutamyl transpeptidase (γ-GT), prothrombin time international normalized ratio (PT INR), albumin, WBC, and C-reactive protein (CRP), were assessed from peripheral blood testing. The cut-off values of biochemical markers, except CRP and serum ferritin, were determined according to the reference ranges of markers. In this study, the cut-off value for CRP was defined as 1.0 mg/L, which is commonly used in systemic inflammation and cancer studies [21, 22]. Serum ferritin was categorized into two groups according to the iron overload criteria suggested by the World Health Organization, which defined low serum ferritin as ≤200 ng/mL in men and ≤ 150 ng/mL in women. A high level of serum ferritin was defined as > 200 ng/mL in men and > 150 ng/mL in women [23].

### Overall survival

Overall survival (OS) was defined as the time from the first visit to the time of death from any cause. When the patient was lost to follow-up or the death was not recorded, the patient was censored. The survival time of censored patients was defined by the period from the first visit to the last day of follow-up or to February 25, 2017, the date on which the survival was investigated.

### Treatment

For the treatment or management of patients, the *Rhus verniciflua stokes* (RVS) extract that has been reported with anticancer effect was used as the anticancer agent in this study. This extract is known to have anti-migration, anti-proliferative, and apoptotic effects [24, 25]. The RVS was extracted via the standardized method with water at 95 °C, concentrated, and lyophilized in powdered form. After removing the toxic allergen urushiol, the major compounds were examined to ensure consistent quality of the extract [26]. A 500 mg of RVS extract was filled in one capsule. Patients received three oral capsules of RVS extract in a day (a total of 1500 mg) as an anticancer agent. In addition, herbal medicine and acupunctures were also used based on the doctors’ judgment and consideration of the patient’s symptoms.

### Statistical analysis

The OS of patients was calculated using Kaplan-Meier estimates, and the differences between the survival data was compared using the log-rank test. If the survival time was incomplete, right censoring was used in survival analysis. Subgroup analysis according to ferritin level was performed using Chi-square tests. Univariate and multivariate analyses using a Cox proportional hazard model were conducted. A multivariate analysis was performed with statistically significant factors from the univariate analysis (*P*-value ≤0.05) and controlling confounding factors, such as age and gender. Pearson correlation coefficient tests were performed to evaluate the relationship between serum ferritin and other biochemical markers. The data were analyzed using SPSS (version 18.0; SPSS Inc., Chicago, IL, USA). A *P*-value ≤0.05 was considered statistically significant.

## Results

A total of 80 patients, comprising 27 (33.8%) females and 53 (66.3%) males, with advanced hepatobiliary cancers were analyzed. The mean age was 59.7 years. The demographic, clinicopathological, and biochemical characteristics are summarized in Table 1. A total of 33 (41.3%) HCC cases, 44 (55.0%) BTC cases, and 3 (3.8%) combined HCC and CC (cHCC-CC) cases were included. The majority of patients (68.8%) had undergone at least one prior treatment. The median time from the diagnosis of stage IV cancer to the first visit for the initiation of KM treatment was 4.4 months (range, 0.1–43.3 months).

**Table 1:** Baseline characteristics in patients with advanced hepatobiliary cancers (*n* = 80)

Variables	Values	Total (n)	Percent (%)
Clinicopathological factors			
Age (years)	< 65 / ≥65	50/30	62.5/37.5
Gender	Female / Male	27/53	33.8/66.3
ECOG-PS	0 / 1 / 2	14/52/14	17.5/65.0/17.5
BMI (kg/m^2^)	< 23 / ≥23	44/36	55.0/45.0
Tumor Type	HCC / BTC / cHCC-CC	33/44/3	41.3/55.0/3.8
Previous anticancer therapy	No / Yes	25/55	31.3/68.8
Prior surgery	No / Yes	44/36	55.0/45.0
Prior chemotherapy	No / 1st line / 2nd line / ≥3rd line	47/17/7/9	58.8/21.3/8.8/11/3
Prior radiotherapy	No / Yes	66/14	82.5/17.5
Prior TACE	No / 1 / 2 / ≥3	60/3/5/12	75.0/3.8/6.3/15.0
Prior RFA	No / Yes	71/9	88.8/11.3
Laboratory factors			
Ferritin (ng/mL)	Low^a^ / High^b^	45/35	56.3/43.8
Hb (g/dL)	Low^c^ / High^d^	50/30	62.5/37.5
Total bilirubin (mg/dL)	≤1.2 / > 1.2	64/16	80.0/20.0
AST (IU/L)	< 40 / ≥40	37/43	46.3/53.8
ALT (IU/L)	< 40 / ≥40	46/34	57.5/42.5
γ-GT (IU/L)	≤66 / > 66	25/55	31.3/68.8
PT INR	≤1.16 / > 1.16	57/23	71.3/28.8
Albumin (g/dL)	< 3.5 / ≥3.5	13/67	16.3/83.8
WBC (×10^3^/μL)	< 10.0 / ≥10.0	70/10	87.5/12.5
CRP (mg/L)	< 10.0 / ≥10.0	40/40	50.0/50.0

*COG-PS* Eastern cooperative oncology group performance status, *BMI* body mass index, *TACE* transarterial chemoembolization, *RFA* radiofrequency ablation, *Hb* hemoglobin, *AST* aspartate aminotransferase, *ALT* alanine aminotransferase, *rGT* gamma-glutamyl transpeptidase, *PT INR* prothrombin time international normalized ratio, *WBC* white blood cell, *CRP* c-reactive protein, *HCC* hepatocellular carcinoma, *BTC* biliary tract cancers, *cHCC-CC* combined hepatocellular-cholangiocarcinoma

^a^ female≤150 male≤200, ^b^ female> 150 male> 200, ^c^ female < 12.0 male < 13.0, ^d^ female ≥12.0 male ≥13.0

**Fig. 2:**
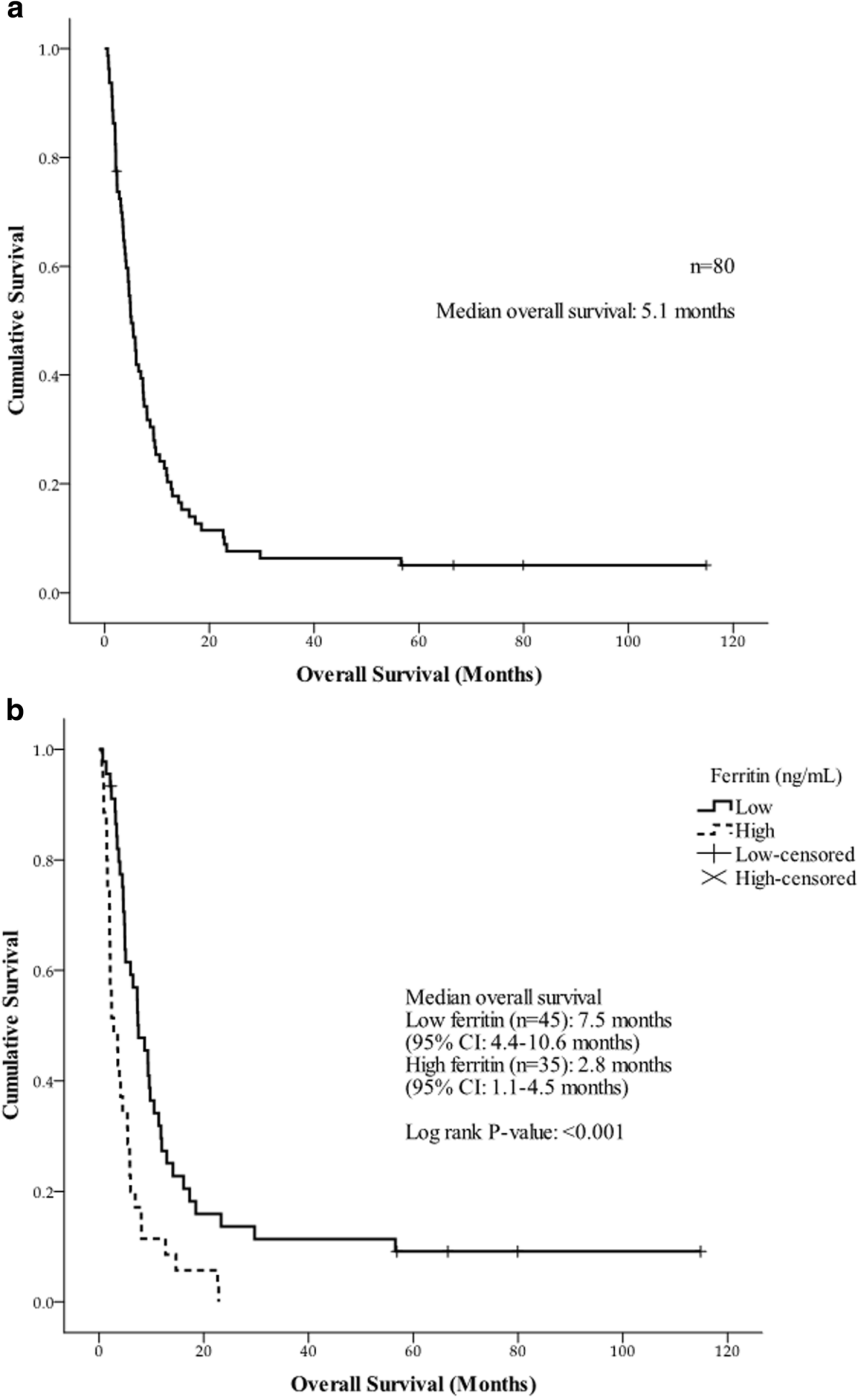
Overall survival of enrolled patients. **a** Overall survival of all patients and **b** Overall survival according to serum ferritin level in advanced hepatobiliary cancers

The median OS of all patients was 5.1 months (range, 0.5–114.9 months) (Fig. 2). The median OS of HCC, BTC, and cHCC-CC was 8.1 months (range, 0.7–114.9 months), 4.5 months (range, 0.5–22.6 months), and 2.1 months (range, 2.0–3.8 months), respectively.

The median OS of the low-ferritin group was 7.5 months (range, 0.7–114.9 months), and the median OS for the high ferritin group was 2.8 months (range, 0.5–22.8 months) (*P* < 0.001) (Fig. 2). Patients with a high serum ferritin showed a significantly shorter survival compared with patients with low serum ferritin.

The low-ferritin group included 45 patients, whereas the high-ferritin group had 35. As shown in Table 2, no statistical differences were noted in age, gender, tumor type, prior treatment, BMI, Hb, total bilirubin, AST, ALT, PT INR, and albumin between the two groups. However, an elevated serum ferritin level was associated with worsening of ECOG-PS (*P* = 0.004) and increasing level of γ-GT (*P* < 0.001), CRP (*P* < 0.001), and WBC count (*P* = 0.013).

**Table 2:** Subgroup analysis according to serum ferritin level (*n* = 80)

Variables	Values	Ferritin (ng/mL)		*P*-value
		Low^a^	High^b^	
		*n* = 45	*n* = 35	
Age (years)	< 65 / ≥65	31/14	19/16	0.181
Gender	Female / Male	15/30	12/23	0.930
ECOG-PS	0–1 / 2	42/3	24/11	0.004
BMI (kg/m^2^)	< 23 / ≥23	23/22	21/14	0.430
Tumor Type	HCC / BTC or cHCC-CC	22/23	11/24	0.116
Previous anticancer therapy	No / Yes	11/34	14/21	0.136
Hb (g/dL)	Low^c^ / High^d^	28/17	22/13	0.950
Total bilirubin (mg/dL)	≤1.2 / > 1.2	37/8	27/8	0.570
AST (IU/L)	< 40 / ≥40	24/21	13/22	0.150
ALT (IU/L)	< 40 / ≥40	27/18	19/16	0.610
γ-GT (IU/L)	≤66 / > 66	22/23	3/32	< 0.001
PT INR	≤1.16 / > 1.16	32/13	25/10	0.980
Albumin (g/dL)	< 3.5 / ≥3.5	7/38	6/29	0.850
WBC (×10^3^/μL)	< 10.0 / ≥10.0	43/2	27/8	0.013
CRP (mg/L)	< 10.0 / ≥10.0	31/14	9/26	< 0.001

*ECOG-PS* Eastern cooperative oncology group performance status, *BMI* body mass index, *Hb* hemoglobin, *AST* aspartate aminotransferase, *ALT* alanine aminotransferase, *γ-GT* gamma-glutamyl transpeptidase, *PT INR* prothrombin time international normalized ratio, *WBC* white blood cell, *CRP* c-reactive protein, *HCC* hepatocellular carcinoma, *BTC* biliary tract cancers, *cHCC-CC* combined hepatocellular-cholangiocarcinoma

^a^ female≤150 male≤200, ^b^ female> 150 male> 200, ^c^ female < 12.0 male < 13.0, ^d^ female ≥12.0 male ≥13.0

The results of the univariate analysis according to demographic and clinicopathological factors are shown in Table 3. The ECOG-PS of 2 compared with ECOG-PS of 0 or 1 (HR = 2.67, 95% CI: 1.44–4.92; *P* = 0.002), and no history of prior treatment (HR = 1.77, 95% CI: 1.08–2.89; *P* = 0.023) were associated with shortened OS. In addition, the OS between tumor types was significantly different. The BTC or cHCC-CC had a significantly increased hazard ratio compared with HCC (HR = 2.50, 95% CI: 1.49–4.19; *P* = 0.001).

**Table 3:** Univariate and multivariate analysis of factors affecting overall survival of patients with advanced hepatobiliary cancers (*n* = 80)

Variables	Values	Univariate		Multivariate	
		Hazard Ratio (95% CI)	*P*-value	Hazard Ratio (95% CI)	*P*-value
Clinicopathological factors					
Age (years)	< 65 vs. ≥65	1.45 (0.91–2.33)	0.121	0.96 (0.56–1.65)	0.880
Gender	Male vs. Female	1.51 (0.93–2.45)	0.096	1.55 (0.86–2.78)	0.144
ECOG-PS	0–1 vs. 2	2.67 (1.44–4.92)	0.002	2.61 (1.26–5.44)	0.010
BMI (kg/m^2^)	< 23 vs. ≥23	0.70 (0.44–1.11)	0.127		
Tumor Type	HCC vs. BTC or cHCC-CC	2.50 (1.49–4.19)	0.001	2.18 (1.15–4.15)	0.018
Previous anticancer therapy	Yes vs. No	1.77 (1.08–2.89)	0.023	1.01 (0.53–1.90)	0.980
Prior surgery	Yes vs. No	0.77 (0.49–1.21)	0.250		
Prior chemotherapy	No vs. Yes	1.31 (0.82–2.08)	0.260		
Prior radiotherapy	No vs. Yes	1.12 (0.62–2.00)	0.710		
Biochemical factors					
Ferritin (ng/mL)	Low^a^ vs. High^b^	2.47 (1.54–3.96)	< 0.001	1.96 (1.03–3.73)	0.041
Hb (g/dL)	High^c^ vs. Low^d^	2.24 (1.35–3.73)	0.002	1.50 (0.81–2.79)	0.200
Total bilirubin (mg/dL)	≤1.2 vs. > 1.2	2.44 (1.37–4.35)	0.002	2.02 (1.00–4.07)	0.051
AST (IU/L)	< 40 vs. ≥40	1.56 (0.99–2.47)	0.058		
ALT (IU/L)	< 40 vs. ≥40	0.85 (0.53–1.36)	0.500		
γ-GT (IU/L)	≤66 vs. > 66	1.98 (1.20–3.25)	0.007	0.91 (0.46–1.80)	0.790
PT INR	≤1.16 vs. > 1.16	1.33 (0.81–2.19)	0.260		
Albumin (g/dL)	≥3.5 vs. < 3.5	2.16 (1.18–3.98)	0.013	1.64 (0.77–3.49)	0.198
WBC (×10^3^/μL)	< 10.0 vs. ≥10.0	2.98 (1.49–5.98)	0.002	1.80 (0.79–4.11)	0.165
CRP (mg/L)	< 10.0 vs. ≥10.0	3.23 (1.99–5.25)	< 0.001	2.12 (1.20–3.76)	0.010

*ECOG-PS* Eastern cooperative oncology group performance status, *BMI* body mass index, *Hb* hemoglobin, *AST* aspartate aminotransferase, *ALT* alanine aminotransferase, *γ-GT* gamma-glutamyl transpeptidase, *PT INR* prothrombin time international normalized ratio, *WBC* white blood cell, *CRP* c-reactive protein, *HCC* hepatocellular carcinoma, *BTC* biliary tract cancers, *cHCC-CC* combined hepatocellular-cholangiocarcinoma, *CI* confidence interval

^a^ female≤150 male≤200, ^b^ female> 150 male> 200, ^c^ female ≥12.0 male ≥13.0, ^d^ female < 12.0 male < 13.0

The results of the univariate analysis according to biochemical factors are shown in Table 3. Biochemical factors were dichotomized either based on the reference values of each marker or based on previous studies [21, 22, 27]. A high serum ferritin level (HR = 2.47, 95% CI: 1.54–3.96; *P* < 0.001), total bilirubin > 1.2 mg/dL (HR = 2.44, 95% CI: 1.37–4.35; *P* = 0.002), γ-GT > 66 IU/L (HR = 1.98, 95% CI: 1.20–3.25; *P* = 0.007), WBC ≥ 10.0 × 10^3^/μL (HR = 2.98, 95% CI: 1.49–5.98; *P* = 0.002), and CRP ≥ 10.0 mg/L (HR = 3.23, 95% CI: 1.99–5.25; *P* < 0.001) were significantly associated with poor survival outcomes. Low Hb level (< 12.0 g/dL for female and < 13.0 g/dL for male) (HR = 2.24, 95% CI: 1.35–3.73; *P* = 0.002) and albumin < 3.5 g/dL (HR = 2.16, 95% CI: 1.18–3.98; *P* = 0.013) were significantly associated with a short OS.

Based on the results of the multivariate analysis, serum ferritin was a significant independent prognostic factor after adjusting for gender, age, ECOG-PS, and other biochemical markers (Table 3). Although age and gender were not significant prognostic factors for survival in the univariate analysis, the multivariate analysis was performed after controlling for age and gender, which influence the level of serum ferritin [13]. A high level of serum ferritin was significantly associated with short survival time (HR = 1.96, 95% CI: 1.03–3.73; *P* = 0.041). Other significant independent prognostic factors for survival were ECOG-PS, tumor type, and CRP. Patients with ECOG-PS of 2 had a significantly shorter survival time than those with ECOG-PS of 0 or 1 (HR = 2.61, 95% CI: 1.26–5.44; *P* = 0.010). The BTC or cHCC-CC had an increased hazard ratio of mortality compared with HCC (HR = 2.18, 95% CI: 1.15–4.15; *P* = 0.018). An elevated CRP was significantly associated with poor survival outcomes (HR = 2.12, 95% CI: 1.20–3.76; *P* = 0.010).

According to correlation analysis, ALT (*r* = 0.23, *P* = 0.037), γ-GT (*r* = 0.23, *P* = 0.038), PT INR (*r* = 0.33, *P* = 0.002), albumin (*r* = − 0.28, *P* = 0.012), WBC (*r* = 0.46, *P* < 0.001), and CRP (*r* = 0.68, *P* < 0.001) were significantly correlated with serum ferritin (Table 4).

**Table 4:** Correlation between serum ferritin and biochemical factors (*n* = 80)

Variables	Correlation coefficients	*P*-value
Hb (g/dL)	−0.13	0.240
Total bilirubin (mg/dL)	0.18	0.117
AST (IU/L)	0.21	0.064
ALT (IU/L)	0.23	0.037
γ-GT (IU/L)	0.23	0.038
PT INR	0.33	0.002
Albumin (g/dL)	−0.28	0.012
WBC (×10^3^/μL)	0.46	< 0.001
CRP (mg/L)	0.68	< 0.001

*Hb* hemoglobin, *AST* aspartate aminotransferase, *ALT* alanine aminotransferase, *γ-GT* gamma-glutamyl transpeptidase, *PT INR* prothrombin time international normalized ratio, *WBC* white blood cell, *CRP* c-reactive protein

## Discussion

Hepatobiliary cancers are fatal cancers usually diagnosed at a progressed stage. Only a minority of patients are eligible for curative treatments [28]. The National Comprehensive Cancer Network recommends sorafenib, a molecular target agent, as a first-line standard treatment for patients with advanced HCC based on two phase III clinical trials [7, 8]. In South Korea, the median OS of patients with advanced HCC who were treated with sorafenib is 4.7 months [29].

For advanced BTC patients with good performance status, gemcitabine and cisplatin combination therapy is the first-line treatment [9]. However, similar to advanced HCC, the available systemic therapy in BTC is also limited, and the median OS of gemcitabine and cisplatin combination therapy is only 9.8 months [30]. In addition, there is no established second-line therapy for disease progression after initial treatment. Therefore, some patients with advanced HCC or advanced BTC who relapsed from the initial treatment or showed a refractory disease status are re-treated with various treatments, participate in clinical trials, or receive the best supportive care. Some patients are reluctant to sequential conventional therapy because of the uncertainty of the benefit and the risk of adverse events of second-line therapy.

In South Korea, KM is typically used for patients with advanced cancer who are not eligible for any conventional standard therapies. In this study, the main herbal medicine prescribed was RVS extract used as the anticancer agent. This extract has been used to treat cancer since the fifteenth century and is known for its antioxidant and anticancer effects. In addition, it has a low cytotoxicity and has been associated with growth inhibition and apoptotic effects in mouse tumorigenic hepatic cells [24, 25, 31–33]. The extract is used in various cancer treatments, long-term complete responses have been reported in two metastatic renal cell carcinomas, and the prolongation of OS with fewer adverse events has been reported in advanced non-small cell lung cancer and pancreatic cancer [34–36]. In hepatobiliary cancer, a patient with recurrent HCC after liver transplantation experienced prolonged survival when treated with RVS [37]. Regarding advanced hepatobiliary cancers, a clinical trial performed with treatment naïve patients reported 7.0 months of median OS [38]. Considering the majority of patients (68.8%) have undergone prior anticancer therapy and the median duration time to initiate RVS extract was 4.1 months after stage IV diagnosis, the 5.1 months median OS in present study could be regarded as a relatively positive clinical outcome.

For patients with advanced hepatobiliary cancers, precise prognosis is vital for deciding on further management because of the aggressive characteristics of advanced cancer. Identifying simple and easily accessible biomarkers for clinical application is necessary. To identify a potential biomarker for predicting survival, this study assessed the significance of serum ferritin as a potential prognostic factor in patients with advanced hepatobiliary cancers.

All 80 patients with advanced hepatobiliary cancers treated with KM only were diagnosed with stage IV disease, and the median time from the diagnosis of stage IV disease to the first visit to the cancer center for the initiation of KM was 4.4 months. The majority of patients (68.8%) had undergone at least one conventional therapy. The median survival of all the patients was 5.1 months, and elevated serum ferritin was significantly correlated with short OS (*P* < 0.001). Based on the results of univariate analyses, ECOG-PS, tumor type, prior treatment, serum ferritin, Hb, total bilirubin, γ-GT, albumin, WBC, and CRP significantly affected OS. In the multivariate analysis controlling confounding factors, serum ferritin was an independent prognostic factor for OS. This finding implies the prognostic impact of serum ferritin regardless of confounding biases, such as age, gender, ECOG-PS, tumor type, prior treatments, and other biochemical markers.

Regarding hepatobiliary cancers, only a few studies surveyed the relationship between serum ferritin level and OS. In HCC patients treated with RFA, high serum ferritin level was a negative risk factor for OS [16]. In patients with intrahepatic cholangiocarcinoma (ICC) undergoing surgical treatments, elevated serum ferritin level was also associated with poor survival outcomes [39]. These studies implied the potential prognostic impact of the serum ferritin level in newly diagnosed or early stage HCC and ICC but not in stage IV hepatobiliary cancers as well as relapsed or refractory disease.

Along with serum ferritin, ECOG-PS, tumor type, and CRP were the significant independent prognostic factors in advanced hepatobiliary cancer patients treated with KM. The ECOG-PS reflects the degree of physical mobility of patients and is repeatedly demonstrated to be a prognostic factor for OS in advanced cancer patients. Similar to previous studies, this study showed the significant relationship between ECOG-PS and the survival outcome [40]. In this study, the OS of advanced BTC and cHCC-CC were shorter than advanced HCC, revealing significantly higher mortality risk [41, 42]. This is the first study comparing the OS of advanced HCC, BTC, and cHCC-CC, and advanced HCC showed significantly longer OS than other tumor types. In addition, the present study also identified CRP as an independent prognostic factor predicting survival which agrees with the findings of previous studies [43, 44].

Ferritin is a 24-subunit protein capable of sequestrating up to 4500 iron atoms in non-toxic form. Ferritin has two types of subunits, namely, light-chain ferritin (L-ferritin) and heavy-chain ferritin (H-ferritin), and they have different functions and characteristics [45, 46]. The ratio of the two differs depending on the sites, and serum ferritin is rich in L-ferritin [47, 48]. L-ferritin concentration in serum is increased in malignancy [47]. The cancer-associated elevation of serum ferritin is most likely caused by an inflammatory state, and a study demonstrated that ferritin is secreted from tumor associated macrophage [49–51]. Due to KM treatment is based on the host rather than tumor, ferritin which is secreted by macrophages and responds to systemic inflammation could be a host based prognostic factor to reflect the status of patients.

Correlation analyses in this study showed significant positive correlations between serum ferritin and inflammation-related biochemical markers, CRP (*r* = 0.68, *P* < 0.001), and WBC (*r* = 0.46, *P* < 0.001) (Table 4). In subgroup analyses, according to serum ferritin level, CRP (*P* < 0.001) and WBC (*P* = 0.013) showed significantly different distribution depending on the serum ferritin level (Table 2). These findings support the correlation between serum ferritin and CRP and WBC in advanced hepatobiliary cancer patients treated with KM. Despite the significant positive correlations with CRP and WBC, only CRP was a significant prognostic marker in multivariate analysis on OS.

Aside from inflammation, damaged hepatobiliary cancer cells may also affect the value of serum ferritin because it was correlated with ALT (*r* = 0.23, *P* = 0.037), γ-GT (*r* = 0.23, *P* = 0.038), PT INR (*r* = 0.33, *P* = 0.002), and albumin (*r* = − 0.28, *P* = 0.012). These results contradict a previous study on HCC that showed no correlation between serum ferritin and transaminase [52]. However, previous studies also showed associations between serum aminotransferase and serum ferritin, which was similar to this study result, suggesting that serum ferritin came from damaged cells, and damaged cells release iron-rich ferritin into the serum [53–55]. Our results also indicate that ECOG-PS, tumor type, and CRP can be considered with serum ferritin in predicting prognosis in patients with advanced hepatobiliary cancers.

This study was limited by its retrospective cohort design and the inclusion of a small number of study participants. Another limitation was the lack of iron-related biomarkers such as serum iron, transferrin, and total iron-binding capacity, because they were not routinely measured in clinical practice. Therefore, well-designed prospective studies are necessary to support the findings of this study.

## Conclusion

This study investigated the clinical significance of serum ferritin as a prognostic factor for survival in advanced hepatobiliary cancer patients treated with KM. The increased serum ferritin was significantly associated with poor survival outcome, showing positive correlation with CRP. After controlling the confounding factors, serum ferritin was identified as an independent prognostic factor for survival in advanced hepatobiliary cancer patients treated with KM. This result implies that serum ferritin plays a significant role in predicting the prognosis for survival in patients with advanced hepatobiliary cancers.

## Abbreviations

ALT: Alanine aminotransferase
AST: Aspartate aminotransferase
BMI: Body mass index
BTC: Biliary tract cancers
cHCC-CC: Combined HCC and CC
CI: Confidence interval
CRP: C-reactive protein
ECOG-PS: Eastern Cooperative Oncology Group Performance Status
Hb: Hemoglobin
HCC: Hepatocellular Carcinoma
HR: Hazard ratio
ICC: Intrahepatic cholangiocarcinoma
KM: Korean medicine
OS: Overall survival
PT INR: Prothrombin time international normalized ratio
RFA: Radiofrequency ablation
RVS: *Rhus verniciflua* stokes
WBC: White blood cell
γ-GT: Gamma-glutamyl transpeptidase
